# Association analysis of *LHCGR* variants and polycystic ovary syndrome in Punjab: a case–control approach

**DOI:** 10.1186/s12902-022-01251-9

**Published:** 2022-12-30

**Authors:** Sukhjashanpreet Singh, Mandeep Kaur, Ratneev Kaur, Archana Beri, Anupam Kaur

**Affiliations:** 1 Department of Human Genetics, Guru Nanak Dev University, Amritsar, Punjab India 143005; 2 Beri Maternity Hospital, Southend Beri Fertility and IVF, Amritsar, Punjab 143001 India

**Keywords:** PCOS, *LHCGR*, rs2293275, rs12470652, BMI, Lipid profile, LH

## Abstract

**Background:**

Polycystic ovary syndrome (PCOS) is an endocrine-metabolic disorder that affects women at their child bearing age. The exact etiology is uncertain, however the involvement of multiple genes and environmental interactions has been proposed for the advancement of PCOS. The aim of present study was to evaluate the association of *LHCGR* variants (rs2293275 and rs12470652) with PCOS in Punjab.

**Methods:**

The present case–control study comprised a total of 743 women (421 PCOS cases and 322 healthy controls). Genotyping was performed using polymerase chain reaction-restriction fragment length polymorphism technique (PCR–RFLP). Biochemical analysis was carried out to measure the levels of cholesterol, High-density lipoprotein (HDL), Low-density lipoprotein (LDL), Very low-density lipoprotein (VLDL), triglycerides, testosterone, luteinizing hormone (LH) and follicle-stimulating hormone (FSH). All the statistical analysis was done using SPSS (version21, IBM SPSS, NY, USA).

**Results:**

The mutant genotype (AA) and mutant allele (A) of rs2293275 conferred 1.7 and 1.3 fold risk, respectively and mutant allele (C) of rs12470652 conferred 2.3 fold risks towards PCOS progression. Levels of cholesterol and triglycerides were elevated and HDL levels were lower in PCOS cases as compared to controls. Total testosterone and luteinizing hormone levels were also found to be higher in PCOS cases.

**Conclusion:**

Our study postulated that *LHCGR* variants are playing a cardinal role in the progression of PCOS and can be used to assess the risk of PCOS in women of reproductive age.

## Introduction

Polycystic ovary syndrome (PCOS) is one of the most common endocrinopathies among women of reproductive age characterized by elevated androgen levels, menstrual irregularities, and/or small cysts in one or both ovaries [1]. PCOS affects 6–26% of women worldwide depending on the diagnostic criteria used and in India prevalence of PCOS is stated to be 11.96% [2, 3]. The Rotterdam criteria (2003) is the most widely accepted criteria for the diagnosis of PCOS [4]. Typical hallmarks include hirsutism, acne, alopecia, loss of scalp hair, dark patches of skin in folds and psychological issues [5]. Although the precise etiology remains unknown, it has been proposed that both genetic and environmental factors may play a cardinal role in the pathogenesis of PCOS [6]. GWAS on Han Chinese [7] and European populations [8] identified the 2p16.3 region (containing *LHCGR* and *FSHR* loci) to be associated with PCOS, with striking differences according to racial background. Several studies have been conducted to analyze the association of susceptible SNPs, which might alter the gene expression or protein function in *LHCGR* and PCOS [9–12].

Luteinizing hormone (LH) triggers follicular growth and ovulation in conjunction with follicle-stimulating hormone (FSH) in ovaries. A surge in LH levels results in ovulation followed by differentiation of the ruptured follicle into the corpus luteum [13]. LH performs its function by binding to its receptor, Luteinizing Hormone/Choriogonadotropin Receptor (LHCGR), which also serves as a receptor for human chorionic gonadotropin (hCG), a glycoprotein hormone that is nearly identical to LH [14].

The *LHCGR* is a single-copy gene consisting of 11 exons and 10 introns located on the short arm of chromosome #2 (2p16.3) [15, 16]. The first 10 exons encode the extracellular domain (ECD), while the last exon encodes a small portion of the ECD, the transmembrane domain and the cytoplasmic C-terminal domain [17]. The mature form of the LHCGR present at the cell surface is a glycoprotein composed of 675 residues with an apparent molecular mass of 85–95 kDa [18–20].

The present study was designed to investigate the association of *LHCGR* rs2293275 and rs12470652 variants (Table 1) with PCOS in the Punjabi population.

**Table 1:** Description of *LHCGR* variants

db SNP ID	Chromosome location	Allele	Location	Substitution type	Molecular consequence	Amino-acid change
rs2293275	Chr2:48,694,236 (GRCh38)	A > G	Exon 10	Non-synonymous	Missense	p.Asn312Ser
rs12470652	Chr2:48,694,299 (GRCh38)	T > C	Exon 10	Non-synonymous	Missense	p.Asn291Ser

## Materials and methods

### Sample collection

In the present study, a total of 743 women comprised of 421 PCOS females and 322 regularly menstruating women having no symptoms of PCOS enrolled as controls. Women diagnosed with PCOS fulfilling the Rotterdam criteria 2003 were recruited from Beri Maternity Hospital, Amritsar, Punjab. The study was approved by the Ethical Committee of Guru Nanak Dev University. Blood samples (5 ml) were collected from November, 2016 to March, 2021. All the information of the subjects was recorded on the predesigned proforma which included demographic information, menstrual and reproductive history, family history and pedigree. Informed consent was taken from each participant before sample collection. Anthropometric measurements such as weight, height and waist-to-hip ratio were also obtained from the included subjects. Cases having the following disorders were excluded: Cushing Syndrome, Congenital hyperplasia, Androgen-secreting tumors and Thyroid dysfunction.

### Biochemical measurements

After sample collection serum was separated from 2 ml of blood by centrifuging the vacutainer at 2000–2500 rpm for 10 min and stored at -20° C till further analysis. Levels of total cholesterol, high-density lipoprotein (HDL) and triglycerides were measured using specific Erba kits on a Biochemical analyzer. Calbiotech’s elisa kits were used to measure the testosterone (T), luteinizing hormone (LH) and follicle stimulating hormone (FSH) levels. To evaluate the proportion of low-density lipoprotein (LDL) and very low-density lipoprotein (VLDL) Friedewald’s formula was used [21].

### DNA extraction

DNA was extracted from 0.5 M EDTA mixed 3 ml peripheral blood using the standard phenol–chloroform method given by Adeli and Ogbonna (1990) with slight modifications [22]. Further, the concentration and quality of extracted DNA were examined using nanodrop.

### Genotype analysis

All the controls were subjected to Hardy–Weinberg equilibrium (HWE) for both SNPs (rs2293275 and rs12470652). Both the genetic variants rs2293275 and rs12470652 appeared to be significantly following HWE. For genotypic analysis, the polymerase chain reaction-restriction fragment length polymorphism (PCR–RFLP) technique was used. Amplification of 111 bp fragment, containing both SNPs, was achieved by using specific primers: forward primer: 5’-CCTCTTCTCTTTCAGACAGA-3’; reverse primer: 5’- CATGCAAATACTTACAGTGTTTTGGTA-3’ [23]. For rs2293275, amplification followed by restriction digestion using *RsaI* (New England Biolabs) enzyme was done at 37° C for 2 h and then electrophoresed on 3.5% agarose gel. After digestion, a product of 111 bp represented homozygous AA genotype, bands of 111 bp, 85 bp and 26 bp signified heterozygous genotype (GA) and bands of 85 bp & 26 bp represented homozygous GG genotype. In the studied population frequency of allele (A) was found to be lower as compared to (G) allele.

Overnight digestion of rs12470652 at 37° C was done using *ApoI* (New England Biolabs) restriction enzyme. After digestion, bands of 88 bp & 23 bp signified wild type genotype (TT), bands of 111 bp, 88 bp & 23 bp represented heterozygous genotype (TC) and band of 111 bp represented mutant genotype (CC). Fragment size analysis was performed on 3.5% agarose gel.

### Statistical analysis

To evaluate the association of *LHCGR* rs2293275 and rs12470652 variants between the cases and controls, chi-square test was performed. Clinical features of both groups were compared using Student’s t-test. One-way analysis of variance (ANOVA) test was used to compare the clinical parameters like cholesterol, triglycerides, etc. The odds ratios (ORs) and confidence intervals (95% CIs) were calculated with MedCalc statistical software. A *p*-value of < 0.05 was accounted for as statistically significant. The power of the study and sample size was determined using the CaTS – Power Calculator which rendered the power > 90% at a confidence interval of 95% and with odds ratio of 1.5 [24]. All the statistical analysis was performed using SPSS (version21, IBM SPSS, NY, USA).

## Result

In this study, the mean ± standard deviation (SD) of age was 24.3 ± 4.89 in cases and 24.6 ± 4.88 in controls. The mean ± SD age at menarche of cases and controls was 12.84 ± 1.33 and 13.13 ± 1.34, respectively. Age at menarche (AAM) was found to be significantly different between the cases and controls (*p* = 0.007). It was observed that women with a sedentary lifestyle were more prone to develop PCOS (*p* = 1.0E^−5^). Women with PCOS showed statistically higher values of BMI, WHR, cholesterol, triglycerides, LDL and VLDL and lower HDL when compared to healthy women. Total testosterone (*p* = 1.0E^−4^) and LH levels (*p* = 6.0E^−3^) were significantly higher in PCOS cases however FSH levels were quite similar among cases and controls (Table 2). Out of 421 PCOS women, 148 (35%) had subtype A, 80 (19%) had subtype B, 64 (15%) had subtype C and 129 (31%) women had subtype D (Table 3).

**Table 2:** Comparison of demographic and biochemical features between PCOS cases and controls

**Variables**	Cases (*n* = 421) (Mean± SD)	Controls (*n* = 322) (Mean ± SD)	**p-value**
Age (year)	24.3 ± 4.89	24.6 ± 4.88	0.49
Age at menarche (year)	12.84 ± 1.33	13.13 ± 1.34	**7.0E**^−3*^
Lifestyle pattern			
Sedentary	315	187	**1.0E**^−5*^
Active	106	135	
BMI (kg/m^2^)	23.9 ± 4.6	22.1 ± 3.39	**1.0E**^−4*^
WHR	0.89 ± 0.066	0.83 ± 0.084	**1.0E**^−3*^
Cholesterol (mg/dl)	176.08 ± 50.31	161.26 ± 40.6	**2.0E**^−5*^
Triglycerides (mg/dl)	161.36 ± 90.57	106.15 ± 63.59	**1.0E**^−5*^
HDL-C (mg/dl)	44.92 ± 13.3	51.18 ± 25.93	**1.0E**^−4*^
LDL-C (mg/dl)	99.26 ± 54.4	88.84 ± 47.49	**7.0E**^−3*^
VLDL-C(mg/dl)	31.89 ± 17.7	21.23 ± 12.71	**1.0E**^−4*^
LH (mIU/ml)	7.9 ± 4.4	5.2 ± 2.7	**6.0E**^−3*^
FSH (mIU/ml)	6.2 ± 2.9	6.1 ± 3.4	0.49
LH/FSH ratio	2.06 ± 3.79	0.89 ± 0.68	0.11
Total testosterone (ng/ml)	0.90 ± 0.33	0.58 ± 0.31	**1.0E**^−4*^

*BMI*  Body Mass Index, *WHR*  Waist-to-Hip Ratio, *HDL*  High-density lipoprotein cholesterol, *LDL-C*  Low-density lipoprotein cholesterol, *VLDL-C*  Very low-density lipoprotein cholesterol, *p < 0.05 Significant

**Table 3:** Distribution of PCOS cases into different phenotypes of Rotterdam criteria

Phenotypes	Cases (%)
A (PCOM + OA + HA)	148 (35)
B (PCOM + HA)	80 (19)
C (OA + HA)	64 (15)
D (OA + PCOM)	129 (31)
Total	421

*PCOM*- Polycystic ovary morphology, *OA*- Oligo-anovulation, *HA*  Hyperandrogenism

The genotypic and allelic frequencies of rs2293275 were found to be statistically significantly different between the cases and controls (*p* = 0.04 and *p* = 0.02, respectively). The mutant genotype (AA), mutant allele (A) and recessive model provided 1.7, 1.3 and 1.53 fold risk, respectively, towards the development of PCOS (Table 4). The genotype frequency of rs12470652 was not significantly different between PCOS cases and controls (*p* = 0.13). However, there was a significant difference of allele distribution between both the groups (*p* = 0.02). The mutant allele (C) conferred 2.3 fold risks towards the PCOS development (Table 4). The one-way ANOVA was used to analyze the LH, FSH, total testosterone and lipid profile with respect to all the genotypes and alleles of both variants in women with PCOS. None of the genotypes showed any significant distribution (Table 5). However, the distribution of VLDL and LH was significantly different between the alleles of rs12470652 (Table 6).

**Table 4:** Distribution of genotypic, allelic frequencies and genetic models of *LHCGR* variants (rs2293275 and rs12470652) between PCOS cases and controls

SNP	Genotype/ Allele	Case (%)	Control (%)	χ^2^ p-value	OR (95% CI)	p-value
**rs2293275**	GG	138 (32.8)	120 (37.2)		Reference	
	GA	162 (38.4)	135 (42)	0.04*	1.04 0.7 to 1.45	0.8
	AA	121 (28.8)	67 (20.8)		1.7 (1.15 to 2.5)	0.006*
	G	438 (52)	374 (58)	0.02*	Reference	
	A	404 (48)	270 (42)		1.3 (1.03 to 1.5)	0.02*
Dominant model	G/G G/A + A/A	138 283	120 203	0.2	- 0.21 0.89 to 1.64	**-** 0.2
Recessive model	G/A + G/G A/A	300 121	255 67	0.01*	- 1.53 1.09 to 2.16	- 0.01*
Heterozygous model	G/G + A/A G/A	259 162	186 136	0.34	- 0.85 0.63 to 1.14	- 0.3
**rs12470652**	TT	402 (95)	314 (97.5)		Reference	
	TC	11 (3)	7 (2.2)	0.13	1.2 (0.47 to 3.2)	0.67
	CC	8 (2)	1 (0.3)		6.2 (0.7 to 50.2)	0.08
	T	815 (97)	635 (98.6)	0.02*	Reference	
	C	27 (3)	9 (1.4)		2.3 (1.09to 5.3)	0.02*
Dominant model	T/T T/C + C/C	402 19	314 8	0.14	- 1.8 (0.8 to 4.2)	- 0.14
Recessive model	T/T + T/C C/C	413 8	321 1	0.04*	- 6.2 (0.77 to 49.7)	- 0.08
Heterozygous model	T/T + C/C T/C	410 11	315 7	0.69	- 1.2 (0.4 to 3.1)	- 0.7

*χ*^2^ – chi square, *OR *Odds ratio, *p < 0.05 Significant

**Table 5:** Distribution of Biochemical and anthropometric parameters concerning genotypes in PCOS cases (mean ± SD)

			rs12470652				rs2293275		
Sr. no		TT	TC	CC	*p*-value	GG	AG	AA	*p*-value
**1**	**Lipid profile**								
	Cholesterol	176.29 ± 51.07	168.59 ± 18.12	175.91 ± 44.46	0.88	169.08 ± 48.6	169.14 ± 45.8	171.58 ± 46.65	0.82
	Triglycerides	161.39 ± 91.83	152.78 ± 63.79	171.85 ± 56.06	0.9	137.09 ± 89.15	134.86 ± 78.81	143.1 ± 87.2	0.58
	HDL	44.81 ± 13.43	46.86 ± 13.19	47.93 ± 10.57	0.71	47.57 ± 14.60	46.77 ± 14.14	49.01 ± 31.47	0.49
	LDL	99.6 ± 55.16	91.16 ± 27.73	93.6 ± 48.04	0.84	94.67 ± 51.25	95.4 ± 48.31	93.98 ± 57.94	0.95
	VLDL	31.87 ± 18.02	30.55 ± 12.75	34.36 ± 11.190	0.89	26.83 ± 17.12	26.96 ± 15.76	28.58 ± 17.42	0.49
**2**	**Anthropometric-Parameters**								
	BMI	24.1 ± 4.5	24.3 ± 4.5	21.6 ± 4.1	0.32	23.6 ± 4.1	23.8 ± 4.6	24.5 ± 4.9	0.31
	WHR	0.85 ± 0.35	0.91 ± 0.30	0.75 ± 0.46	0.63	0.83 ± 0.37	0.82 ± 0.38	0.9 ± 0.29	0.14
**3**	**Hormonal parameters**								
	Total testosterone	0.83 ± 0.3	0.38 ± 0.06	0.58 ± 0.1	0.18	0.76 ± 0.36	0.88 ± .32	0.72 ± 0.27	0.31
	LH	7.4 ± 4.1	14 ± 5.6	9.9 ± 5.2	0.08	6.7 ± 3.1	6.92 ± 3.3	9.36 ± 5.5	0.23
	FSH	6.4 ± 3.17	7.2 ± 3.38	9.14 ± 2.4	0.67	6.08 ± 2.5	6.8 ± 3.02	6.3 ± 3.5	0.84
	LH/FSH	1.53 ± 1.5	2.43 ± 1.9	1.08 ± 1.4	0.71	1.19 ± 0.57	1.1 ± 0.52	2.2 ± 2.2	0.1

One way anova, *p < 0.05 Significant

**Table 6:** Distribution of Biochemical and anthropometric parameters concerning alleles in PCOS cases (mean ± SD)

	rs12470652			rs2293275		
	**T**	**C**	**p-value**	**G**	**A**	**p-value**
**Lipid profile**						
Cholesterol	176.08 ± 50.47	171.67 ± 31.06	0.71	175.22 ± 51.2	176.51 ± 49.8	0.75
Triglycerides	161.16 ± 91.15	160.81 ± 59.8	0.98	159.16 ± 90.27	160.5 ± 86.6	0.83
HDL	44.86 ± 13.41	47.31 ± 11.85	0.57	45.35 ± 14.41	44.4 ± 12.37	0.39
LDL	99.37 ± 54.6	92.1 ± 36.42	0.32	98.54 ± 55.6	100 ± 53.1	0.74
VLDL	31.84 ± 17.89	32.1 ± 11.95	0.006*	31.32 ± 17.31	32.1 ± 17.45	0.59
**Obesity-related parameters**						
BMI	24.04 ± 4.5	23.1 ± 4.5	0.42	23.79 ± 4.4	24.1 ± 4.7	0.33
WHR	0.85 ± 0.35	0.84 ± 0.37	0.91	0.828 ± 0.37	0.85 ± 0.35	0.37
**Hormonal parameters**						
Total testosterone	0.8 ± 0.31	0.45 ± 0.12	0.06	0.80 ± 0.3	0.85 ± 0.32	0.56
LH	7.8 ± 4.47	14.2 ± 4.26	0.02*	8.1 ± 4.5	6.9 ± 3.2	0.93
FSH	6.4 ± 3.1	7.8 ± 2.6	0.46	6.6 ± 3.2	6.7 ± 2.8	0.29
LH/FSH	1.58 ± 1.5	1.98 ± 1.5	0.66	1.62 ± 1.4	1.12 ± 0.52	0.17

One way anova, *p < 0.05 Significant

## Discussion

The present study focused on the association of *LHCGR* variants (rs2293275 and rs12470652) with PCOS. In this cohort study, 421 women with PCOS and 322 women without PCOS were selected and no significant difference of age was found between cases and controls as both the groups were age-matched (Table 2). However, a study done by Jamil and colleagues found a statistically significant difference in ages between PCOS women and controls [25]. In our study, significant difference for AAM was found between PCOS cases and controls (*p* = 0.007). A similar trend for AAM was found in a South Indian case–control study with *p* < 0.05 [26]. In contrast, Dasgupta and Reddy found no significant difference in AAM between cases and controls (*p* = 0.852) [27].

Waist-to-hip ratio (WHR) helps identify potential health risks associated with being overweight and obese. When most of the fat deposition is around the waist instead of the hips, the risk for metabolic disorders like heart disease and type 2 diabetes is augmented. In the present study, WHR was found to be highly significant (*p* = 0.001) (Table 2). However, a study done on Polish women by Kałuzna and colleagues did not find any difference of WHR between both the groups [28].

Obesity is recognized as a risk factor for various metabolic disorders. BMI is traditionally the most widely used measure of obesity; thus predicts the risk for metabolic syndrome [29]. High adipose mass is associated with the increased production of aromatase, leptin and insulin resistance and dyslipidemia, all of which result in tissue damage. BMI was found to be a factor that provided a significant risk of PCOS with *p* = 1.0E^−4^ in the present study (Table 2). Another North Indian study also conferred that overweight/obese women have a high prevalence of classic phenotype of PCOS [30]. Similar results were also found in the Chinese population for BMI [31].

Physical activity is one of the major causes that affect the values of BMI. Women with a sedentary lifestyle are more susceptible to develop obesity which further gives rise to metabolic ailments like type 2 diabetes, cardiovascular diseases and nonalcoholic fatty liver disease [32]. In our study, a significant association was found between physical activity and the development of PCOS. It was observed that women with a sedentary lifestyle were more prone to develop PCOS (*p* = 0.00001) as compared to physically active women (Table 2). Our findings were supported by a study on women in Greece, which concluded that PCOS girls were less engaged in physical activity than healthy individuals [33]. Chau and co-workers performed a meta-analysis and came up with the results supporting the hypothesis that a sedentary lifestyle is significantly associated with the greater risk of all-cause mortality [34]. Contrary to these studies, Lin et al. (2021) found no significant association of physical activity between PCOS cases and controls in United States [35]. In our study, we found a significant association of abnormal lipid profile values in PCOS cases. Levels of cholesterol (*p* = 0.00002), triglycerides (*p* = 0.00001), LDL (*p* = 0.01) and VLDL (*p* = 0.00001) were higher whereas HDL (*p* = 0.0001) levels were low in PCOS as compared to healthy individuals (Table 2). Our results were supported by the other studies on the different populations across the world, [36–39]. Another study from the United States delivered a high prevalence of dyslipidemia in women with PCOS [40].

Exon 10 of *LHCGR* contains rs2293275 and rs12470652 SNPs that lead to changes in the amino acids at positions 312 and 291, respectively. These SNPs may have subtle effects on the LHCGR's sensitivity to LH. This potential is increased by the exon 10 polymorphisms’ proximity to glycosylation sites, which are critical for the stability, trafficking, and expression of the G-protein-coupled receptor superfamily on the cell surface [41]. In the present study, the distribution of genotypic and allelic frequencies was evaluated and compared between both groups. Our study concluded statistically significant association of these two variants with PCOS. A variant rs2293275 was observed significantly associated with increased risk of PCOS. The carriers of mutant genotype (AA) and mutant allele (A) were 1.7 and 1.3 times more susceptible to develop PCOS, respectively. Our results were supported by a study conducted on Sardinian population, which revealed that the presence of at least one 312 N allele provides significant PCOS risk (OR, 2·04; 95% CI, 1·32–3·14; v2, 10·47; *P* = 0·001) and homozygosity for 312 N variant was conferring 2.7-fold increased risk of developing PCOS [23]. Another group evaluated this variant in a case–control study of Egyptian women and they observed the frequency of wild genotype (GG) was 47%, heterozygous (GA) was 27% and mutant genotype (AA) was 26% in women with PCOS, while in controls 70% were wild type and 30% were heterozygous (OR: 2.25; CI: 1.16–4.386; p value = 0.012). They concluded that individuals who were homozygous for this variant were more susceptible to developing PCOS than controls (OR: 1.80; CI: 1.54–2.09; p-value < 0.001) [9]. In a South Indian study, Thathapudi and colleagues demonstrated an association between rs2293275 and PCOS. They found that the G allele was more prevalent in PCOS cases (0.60) as compared to controls (0.49) (OR: 1.53; CI: 1.16–2.01; p-value = 0.002) and carriers of the GG genotype were significantly predisposed to PCOS progression (OR: 3.36, CI: 1.96–5.75; p-value < 0.0001) [11]. A significant association between rs2293275 and PCOS was also shown by a recent study performed on Kashmiri women. They found a significantly higher frequency of homozygous (AA) and heterozygous (GA) genotypes in cases as compared to controls and observed women carrying either GA or AA genotypes were at higher risk of developing PCOS (OR = 10.4, *p* < 0.0001; OR = 7.73, *p* = 0.02 respectively) (Makhdoomi et al*.,* 2022) [42]. A study conducted on the Jordanian population showed a significant difference in the frequency of heterozygous (AG) genotype between cases and controls (p-value < 0.05) (Atoum et al*.,* 2022) [43]. In contrast, a study on Caucasians showed no association between rs2293275 and PCOS [44]. Another study conducted on Sri Lankan women observed no significant difference in genotypic distribution between cases and controls and it also suggests that this variant is most unlikely to be involved in the pathogenesis of PCOS [45]. In a pilot study of 98 unrelated Colombian women, no significant association was found between rs2293275 and PCOS (Alarcón-Granados et al*.,* 2022) [46]. The variant rs12470652 resulting in an amino acid substitution affecting glycosylation which proposes that LH receptor might be more active [41]. The genotypic frequency was not significantly different between cases and controls in present study. However, distribution of allele frequency was found to be significantly different between both groups and it was observed that the mutant allele (C) conferred 2.3-fold risk to PCOS progression. In contrary to our results, Capalbo et al. observed no differences in the distribution of both genotypic and allelic frequencies in Sardinian population [23]. In another study conducted by Valkenburg et al. found no significant differences in the distribution of genotype and allele frequencies in both groups [44].

Our study showed that none of the genotypes rs2293275 and rs12470652 were significantly associated with dyslipidemia and the influence of genotypes on hormonal levels and anthropometric parameters was also not seen (Table 5). However, the significant impact of minor allele of rs12470652 variant on VLDL and LH levels was observed (*p* = 0.006 and *p* = 0.02, respectively) (Table 6). Our results were supported by a study conducted by Valkenburg and colleagues [44]. Contrary to our findings Thathapudi et al. found a significant association of the GG genotype of rs2293275 with BMI, WHR, LH and LH/FSH ratio in PCOS cases [11].

Relative risk was calculated for alleles of *LHCGR* variants between different phenotypes of PCOS and controls. Both variants rs2293275 and rs12470652 appeared to contribute differently to each subtype based on genotype–phenotype correlation analysis. The variant rs2293275 was found to link to specific subtype such as oligo-anovulation, whereas rs12470652 might be exaggerating classical features of PCOS as a variant conferring 2.4, 3.3 and 2.7 folds risk towards subtype A, C and D respectively. The correlation study suggested that genetic background might have an impact on particular subtype and supports the PCOS diagnosis. The limited number of cases in each phenotype group may compromise the statistical power, therefore larger-scale research is required to support this notion (Table 7).

**Table 7:** Distribution of alleles among different phenotypes of PCOS

		**Cases**	**Controls**	**p-value**	**Relative Risk**	**CI (95%)**
**rs2293275 (G/A)**						
**Phenotype A**						
	G	159	374	0.21	1.1	0.95 to 1.3
	A	137	270			
**Phenotype B**						
	G	86	374	0.3	1.1	0.9 to 1.3
	A	74	270			
**Phenotype C**						
	G	62	374	0.04*	1.23	1.02 to 1.5
	A	66	270			
**Phenotype D**						
	G	131	374	0.04*	1.17	1.001 to 1.4
	A	127	270			
**rs12470652 (T/C)**						
**Phenotype A**						
	T	286	635	0.04*	2.4	0.99 to 5.8
	C	10	9			
**Phenotype B**						
	T	156	635	0.32	1.8	0.55 to 5.7
	C	4	9			
**Phenotype C**						
	T	122	635	0.01*	3.3	1.21 to 9.2
	C	6	9			
**Phenotype D**						
	T	251	635	0.02*	2.7	1.13 to 6.6
	C	10	9			

^*^p < 0.05 Significant

## Conclusion

This is the first study from Punjab carried out to investigate the possible association of exon 10 variants of *LHCGR* gene for the development of polycystic ovary syndrome. Both the SNPs rs2293275 and rs12470652 were found to be playing a significant role in the pathogenesis of PCOS in our population. Our study produced baseline data on PCOS genetics, however, large-scale studies are needed to narrow down that how these polymorphisms are affecting the susceptibility of women towards PCOS. 

## Data Availability

The data used in present study are available by corresponding author on request.

## Abbreviations

PCOS: Polycystic Ovary Syndrome
GWAS: Genome Wide Association Studies
LHCGR: Luteinizing Hormone/Choriogonadotropin Receptor
FSHR: Follicle Stimulating Hormone Receptor
SNP: Single Nucleotide Polymorphism
LH: Luteinizing Hormone
hCG: Human Chorionic Gonadotropin
ECD: Extracellular Domain
kDa: Kilodalton
ml: Milliliter
HDL: High-density lipoprotein
T: Testosterone
FSH: Follicle Stimulating Hormone
LDL: Low-density lipoprotein
VLDL: Very low-density lipoprotein
DNA: Deoxyribonucleic acid
EDTA: Ethylene diaminetetraacetic acid
HWE: Hardy–Weinberg Equilibrium
PCR: Polymerase chain reaction
RFLP: Restriction fragment length polymorphism
bp: Base pair
ANOVA: Analysis of Variance
OR: Odds ratios
CI: Confidence intervals
SD: Standard deviation
AAM: Age at menarche
BMI: Body mass index
WHR: Waist-to-hip ratio
PCOM: Polycystic ovary morphology,
OA: Oilgo-annovulation,
HA: Hyperandrogenism
